# A new look at cerebrospinal fluid circulation

**DOI:** 10.1186/2045-8118-11-10

**Published:** 2014-05-01

**Authors:** Thomas Brinker, Edward Stopa, John Morrison, Petra Klinge

**Affiliations:** 1 Department of Neurosurgery, The Warren Alpert Medical School of Brown University, Rhode Island Hospital, 593 Eddy Street, Providence, RI 02903, USA

**Keywords:** Cerebrospinal fluid circulation, Astrocyte, Aquaporin, Blood brain barrier, Virchow Robin space

## Abstract

According to the traditional understanding of cerebrospinal fluid (CSF) physiology, the majority of CSF is produced by the choroid plexus, circulates through the ventricles, the cisterns, and the subarachnoid space to be absorbed into the blood by the arachnoid villi. This review surveys key developments leading to the traditional concept. Challenging this concept are novel insights utilizing molecular and cellular biology as well as neuroimaging, which indicate that CSF physiology may be much more complex than previously believed. The CSF circulation comprises not only a directed flow of CSF, but in addition a pulsatile to and fro movement throughout the entire brain with local fluid exchange between blood, interstitial fluid, and CSF. Astrocytes, aquaporins, and other membrane transporters are key elements in brain water and CSF homeostasis. A continuous bidirectional fluid exchange at the blood brain barrier produces flow rates, which exceed the choroidal CSF production rate by far. The CSF circulation around blood vessels penetrating from the subarachnoid space into the Virchow Robin spaces provides both a drainage pathway for the clearance of waste molecules from the brain and a site for the interaction of the systemic immune system with that of the brain. Important physiological functions, for example the regeneration of the brain during sleep, may depend on CSF circulation.

## Introduction

The anatomy of the cerebrospinal fluid (CSF) system includes the cerebral ventricles as well as the spinal and brain subarachnoid spaces, cisterns and sulci. The traditional understanding of CSF physiology assumes that 80% of CSF is secreted by the choroid plexus into the ventricular cavities. Other structures, e.g. the brain parenchyma, add the remaining 20%. The rate of CSF formation in humans is 0.3 – 0.4 ml min^-1^, and the total CSF volume is 90 – 150 ml in adults. It is also believed that CSF circulates through the ventricles, the cisterns, and the subarachnoid space ultimately to be absorbed into the venous blood at the level of the arachnoid villi. Minor portions of CSF may be drained into the cervical lymphatics that run via the perineural spaces of the cranial nerves. Traditionally, the circulatory character of CSF flow is accepted and coined as the "third circulation" (see for example [1-5]).

The traditional understanding of CSF physiology is mainly based on animal experimentation, which was criticized as early as 1947. It was argued that neuropathological observations fail to support the experimentally-based hypothesis that the origin of the cerebrospinal fluid is largely from the choroid plexus [6]. Recent research challenges significant aspects of the classical model and the circulatory nature of the CSF flow has been questioned. Specific aspects now being reconsidered include the rate and site of CSF formation and absorption [7-10]. This review reexamines key developments that have led to the traditional concept of CSF physiology and introduces new findings that enhance our current understanding. Novel insights from molecular and cellular biology as well as neuroimaging research have shown that CSF physiology is much more complex than previously recognized.

## Review

### Traditional understanding of CSF physiology

#### CSF formation

Most CSF is formed in the cerebral ventricles. Possible sites of origin include the choroid plexus, the ependyma, and the parenchyma [2]. Anatomically, choroid plexus tissue is floating in the cerebrospinal fluid of the lateral, third, and fourth ventricles. This tissue is well perfused by numerous villi, each having a central capillary with fenestrated endothelium. A single layer of cuboidal epithelium then covers each of these vessels. This unusual cellular anatomy forms the blood CSF barrier characterized by tight junctions at the apical end of the choroid epithelial cells rather than at the capillary endothelium within each villus [2,11,12].

Due to its glandular appearance and ventricular location, the choroid plexus has been suggested to be the major site of CSF secretion. This view was mainly based upon the historical canine experiments of Dandy. In these experiments the foramen of Monro was occluded and a choroid plexectomy of one lateral ventricle was performed. The authors reported collapse of the ventricle without choroid plexus and dilatation of the other ventricle [13]. They concluded: "From these experiments we have the absolute proof that cerebrospinal fluid is formed from the choroid plexus. Simultaneously it was proven that the ependyma lining the ventricles is not concerned in the production of cerebrospinal fluid" [14]. Interestingly, the experiments of Dandy were based upon observations from only a single dog [1]. Furthermore, the experiments could not be reproduced by others [15-17].

There were two other sets of experiments that were thought to be "crucial" in support of Dandy’s thesis [1]: First, the hematocrit of the choroid plexus blood was found to be 1.15 times greater than of that of the systemic arterial blood. From this value and the estimated arterial blood flow through the choroid plexus, a CSF secretion rate was calculated that came very close to the estimated rate of total CSF absorption [18]. Second, these findings were substantiated by concordance with experiments in which the CSF production rate was assessed in the isolated and extracorporally-perfused choroid plexus [19-22]. These experiments, however, were criticized because of inherent large errors possible in the experimental technique since the various preparations all required considerable operative manipulations [1,2,11]. Furthermore, other experimental studies, including those with radioactive water provided evidence that at least some CSF must come from a source other than the choroid plexus, presumably the brain tissue itself [23-25]. From perfusion studies performed on isolated regions of the ependymal surface it was calculated that nearly 30% of the total CSF production may come from the ependyma [26]. An even higher rate of ependymal fluid secretion was derived from experiments investigating spinal cord ependyma [27]. Again, these experiments were criticized because of the "drastic experimental procedures" used. It was concluded that "it may be wise to reserve final judgment on this question" [11]. The capillary-astrocyte complex of the blood–brain barrier (BBB) has been implicated as an active producer of brain interstitial fluid (ISF). The ISF secreted at the blood–brain barrier is coupled with shifts of extracellular fluid between brain and CSF, eventually leading to the net formation of CSF [28,29]. The rate of ISF formation was estimated from the clearance rate of tracer substances, which were injected into the brain parenchyma. It was assumed that the rate of clearance provides an estimate of the rate of ISF secretion at the blood–brain barrier. The calculated rate of formation was substantially lower (1/100 when normalized for barrier surface area) than the choroid plexus production rate [30]. Accordingly, even a recent review concluded that "the working hypothesis that the BBB is a fluid generator, although attractive, needs substantiation" [4].

#### CSF absorption

Historically, the absorption of CSF into the circulating blood is most notable across the arachnoid villi [3,31,32]. It was stated: "From a purely anatomical point of view, these arachnoid villi are obvious regions for the drainage of CSF into the vascular system…" (page 486 in [33]). The notion of the arachnoid villi being the major site of CSF absorption is actually based on the early experiments of Key and Retzius who injected colored gelatin into the CSF space of human cadavers. They reported the distribution of the dye throughout the entire CSF system and its passage across the arachnoid villi into the venous sinuses [34]. However, their results were questioned since the dye was injected at a pressure of up to 60 mmHg. It was suggested that the high pressure during the dye injection could cause rupture of the arachnoid villi and absorption into the sinuses [35]. Therefore, Weed performed dye injection experiments at pressures of only 9–13 mmHg that also attempted to determine whether or not the injected dye particles themselves could obstruct the normal drainage pathways. Isotonic solutions of non-toxic dyes (ammonium citrate and potassium ferrocyanide) were infused that precipitated granules of Prussian blue before the animals were intravitally fixed with acidified formalin. Weed reported the distribution of the dye particles throughout the entire CSF space, filling the arachnoid villi along the sagittal sinus, eventually invading the dural wall of the sinus. Notably, only some granular material was found in the lumen of the sinus [35,36]. The authors also stated as another important result: "No evidence has been afforded in our observations of the escape of cerebrospinal fluid into cerebral veins or capillaries" [37]. Weed’s findings formed the basis for the principal understanding of CSF absorption, accepted by the majority of researchers today. Weed performed numerous pilot experiments in his effort to identify a dye solution that was best suited for his studies: "Many solutions were tried, but all proved unsatisfactory because of their toxicity or their diffuse tissue staining" [36]. One could argue, therefore, that Weed inadvertently excluded those solutions in which the absorption of CSF throughout the entire brain parenchyma would have been the result. Electron microscopy studies performed on arachnoid villi revealed a pressure-sensitive vacuolation cycle of pores, which act as one-way valves and allow for the transcellular bulk transport of fluid [38,39]. Extracorporal perfusion of excised dura demonstrated the passage of particles up to the size of erythrocytes [40].

Considerable portions of CSF may be absorbed into the cervical lymphatics [2]. The perineural subarachnoid space of cranial nerves, which is connected to the cranial CSF space, was suggested as a pathway for the drainage of CSF into the lymphatics of the extracranial soft tissue at the skull base [2]. Though it is obvious that CSF drains into the lymphatics, the physiological significance of this CSF absorption route is still a matter of debate. Five hours after the injection of albumin dye into the CSF space of rabbits only 5% is typically seen draining into the cervical lymph nodes [41]. This finding led to the conclusion that only a small fraction of CSF drains via the lymphatic channels. However, in the same period of time only 14% of the injected dye was found in the blood, revealing that lymphatic channels contributed to 26% of the tagged protein that had left the central nervous system and entered the blood stream [2]. Following the infusion of iodine-labeled serum human albumin into sheep, it was determined that 40–48% of total volume of CSF is absorbed from the cranial compartment by extracranial lymphatics [42]. A lymphatic drainage fraction of 50% was estimated from the injection of radioiodinated albumin (RISA) into the brain of rabbits. Interestingly much RISA was drained via the cerebral perivascular spaces as well as by the passage from the subarachnoid space of olfactory lobes into the submucosal spaces of the nose (and thus to the lymphatics) [43]. Intravital microscopy of the exposed cervical lymph nodes during the cisternal infusion of ink revealed that particle movement was dependent on the respiratory cycle: during inspiration the speed of particle movement was 10–20 mm s^-1^, while no movement was observed during the expiration phase [44]. It is important to note that the CSF and ISF spaces communicate with the cervical lymphatics via two anatomically different routes, i.e. the perineural subarachnoid space of cranial nerves and a "prelymphatic" pathway along the arterioles and arteries of the brain (see discussion below, reviewed by [45]).

Extracranial organs feature fluid exchange across the capillary bed that is driven by hydrodynamic and osmotic pressure gradients. However, absorption of CSF into cerebral capillaries has been disputed because it was thought that the absorption of CSF is not dependent on osmotic forces. This notion was based on experiments in which dextran solutions of different osmolality were infused into the ventricles of cats at a constant pressure of 27 mmHg. The measured infusion rate, which should equal the CSF absorption rate, decreased by the same extent. The decrease of the absorption rate was explained by the increased CSF viscosity [33]. Interestingly, a more recent animal study failed to reproduce these earlier experiments, since it was shown that ^3^H_2_O from the bloodstream enters osmotically loaded cerebrospinal fluid significantly faster [46]. Since, historically, osmolality was assumed to not be relevant for CSF absorption, hydrodynamic pressure gradients would be the only driving forces for CSF drainage into the brain capillaries and post-capillary venules. It was also assumed that any absorption would require a CSF pressure higher than the intravascular pressure and that this would cause the collapse of the vessels and prevent absorption of CSF [2,47]. These statements from the 1970s and 1980s were actually defining the understanding of CSF physiology for decades until BBB and aquaporin (AQP) studies clearly indicated the involvement of osmotic forces in brain water homeostasis (for discussion see below).

#### Assessment of CSF formation and absorption rate

In 1931, Masserman calculated the rate of CSF formation in patients by measuring the time needed for the CSF pressure to return to its initial level following drainage of a standard volume of CSF by lumbar puncture [48]. After drainage of 20 to 35 mL of CSF, pressure was restored at a rate of about 0.32 ml min^-1^. The validity of results obtained in this way was criticized because the Masserman technique assumes that neither formation nor absorption rates are changed by alterations in pressure. However, the absorption of CSF varies greatly with changes in intracranial pressure [49,50]. Modifications of the Masserman technique applied sophisticated infusion and drainage protocols, which recorded and controlled the CSF pressure during the measurement period (see for example [51]). Despite numerous research efforts, more sophisticated experimental protocols did not yield CSF formation rates that differed from earlier work.

The ventriculo-cisternal perfusion ("Pappenheimer") technique represents a more quantitative approach for the assessment of CSF formation rate. Inulin or other macromolecules, which pass through the ventricular space without being absorbed, are infused at a constant rate into the cerebral ventricles. CSF formation is calculated from the measurement of the extraventricular (cisternal or spinal) CSF concentration of inulin. It is assumed that any dilution of inulin between the inflow cannula and outflow cannula results from the admixture of freshly formed CSF. In addition, the test procedure allows for the calculation of the CSF absorption rate from the clearance of inulin at the extraventricular site (in animals the cisterna magna, in man the lumbar space) [49]. An important disadvantage was that the procedure was difficult to apply in clinical settings because of its invasiveness: The hour long infusion required both a ventricular and extraventricular CSF catheter. Also, both infusion rate and infused volume exceeded the physiological range of CSF flow by far. Despite these obstacles, clinical measurements were performed in brain tumor patients who received ventricular catheters for chemotherapy purposes: In patients (9–61 years old) the average flow rate was 0.37 ml min^-1^, the maximum absorption capacity was 1.3 ml.min^-1^[52]. These results were confirmed in children with brain tumors [53]. Furthermore, similar data are available from hydrocephalus patients [54]. Though more precise, the ventriculocisternal or ventriculolumbar perfusion techniques yielded results remarkably close to those assessed by the Masserman technique [2]. Findings from both the Masserman and the Pappenheimer techniques were supported by neuroradiological investigations applying serial CT scans to assess the ventricular washout of metrizamide, a water soluble contrast media. The rate of right lateral ventricular CSF formation ranged from 0.0622 to 0.103 ml min^-1^[55,56]. Hence, the assessment of the CSF formation and absorption rates remains a matter of debate even today. It has been suggested that a method that is less invasive than the Pappenheimer method (ventriculo-cisternal perfusion) and more reliable than the Masserman method is sorely needed [50].

#### CSF circulation

The concept of the "third circulation" suggesting that CSF flows through the ventricles, cisterns and subarachnoid space (SAS) and is reabsorbed into the blood at the arachnoid villi, was introduced by Cushing in 1926 [57,58]. This notion was a radical departure from the contemporary view that the CSF moved by ebb and flow [1]. Since Cushing, the circulatory, bulk flow character of the CSF system has remained unquestioned by the majority of researchers. Even recent reviews assume a directed CSF circulation through the ventricles and the subarachnoid space toward the arachnoid villi [1,5,32]. Nevertheless, as will be discussed below, this understanding of CSF circulation appears to be a rough simplification of a much more complicated situation. This especially holds true for the circulation of CSF along the Virchow–Robin spaces (VRS). The current classical view assumes that CSF flow along the VRS is slow and physiologically not important [4,5].

#### Virchow-Robin space circulation

Anatomically the VRS refers to a histologically-defined space, which surrounds blood vessels (arterioles and venules) when penetrating from the subarachnoid space into the brain tissue. Originally, it was thought that the VRS is connected to the subarachnoid space, allowing for a free fluid communication. It was suggested that interstitial fluid may be outwardly drained along these pathways into the SAS and eventually towards the arachnoid villi [35]. Later this concept was questioned on the basis of light microscopic examinations, which depicted perivascular spaces as cul-de-sacs, open to the subarachnoid space but closed towards the parenchyma and therefore not a channel for flow [59]. The first systematic electron microscopic study of blood vessels entering the cerebral cortex confirmed this view. In addition it was reported that small arterioles entering the cortex carry with them (to the point at which they become capillaries) an extension of the subarachnoid space [60]. Actually, these findings, showing the obliteration of the VRS at the capillary bed, led to the rejection of the earlier theories on the existence of a perivascular CSF circulation. As discussed by others [61], these morphological findings eventually supported the general belief that the interstitial fluid (ISF) is stagnant in the central nervous system.

**Figure 1:**
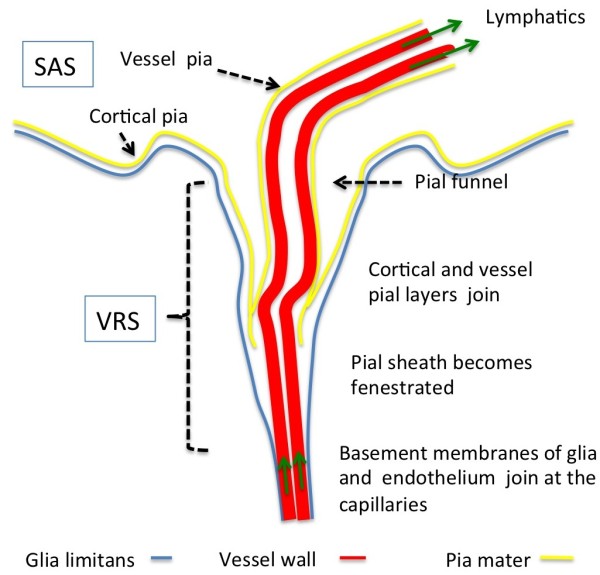
**Morphology of Virchow Robin and perivascular spaces.** Delineated by basal membranes of glia, pia and endothelium, the Virchow Robin space (VRS) depicts the space surrounding vessels penetrating into the parenchyma. The VRS is obliterated at the capillaries where the basement membranes of glia and endothelium join. The complex pial architecture may be understood as an invagination of both cortical and vessel pia into the VRS. The pial funnel is not a regular finding. The pial sheath around arteries extends into the VRS, but becomes more fenestrated and eventually disappears at the precapillary section of the vessel. Unlike arteries (as shown in this figure), veins do not possess a pial sheath inside the VRS. ISF may drain by way of an intramural pathway along the basement membranes of capillaries and arterioles into the lymphatics at the base of the skull (green arrows). It should be noted that the figure does not depict the recently suggested periarterial flow from the SAS into the parenchyma and an outward flow into the cervical lymphatics along the veins (for discussion see text "Current research"). Also, it is still a matter of debate whether the Virchow Robin space, extending between the outer basement membrane of the vessel and the glia, represents a fluid-filled open space (see text). VRS: Virchow Robin space, SAS: subarachnoid space.

The current understanding of the microscopic anatomy of the VRS is more complex (Figure 1). Actually, its fine structure is built upon endothelial, pial, and glial cell layers, each of them delineated by distinct basement membranes [62-64]. The glial membrane (glia limitans) covering the brain parenchyma forms the outer wall of the VRS [65]. At the capillary bed, the basement membrane of the glia fuses with the outer vascular membrane thereby occluding the Virchow-Robin space [66,67]. Arterial and venous vessels running within the cortical subarachnoid space are covered with a pial cell layer, which ensheaths the vessels. The pial sheath creates a space next to the vessel wall, which is referred to as perivascular space (PVS) [68]. At the site of the entrance of the cortical vessels into the VRS, their pial sheath joins with the pial cell layer covering the brain surface forming a funnel like structure, which accompanies the vessels into the VRS though for a short distance only [69,70]. However, the pial sheath of the arterial, but not venous, vessels extends into the VRS. Near the capillary bed, the pial sheath becomes more and more fenestrated and leaky [68]. It is important to note that the nomenclature is not used consistently. Some authors use the terms "Virchow Robin space" and "perivascular space" as synonyms [71], while others use the terms to name different spaces as discussed above [72].

Ultrastructural electron microscopic studies agree that pial membranes separate the VRS from the cortical subarachnoid space [65,68,70]. Since electron microscopy of human brain specimens shows that the VRS and the PVS are collapsed [68], it is a matter of debate whether these histologically-characterized compartments are actually open or just potential spaces. However, studies in rodents have demonstrated the VRS filled with fluid, electron microscopic dense material [70], macrophages and other blood born inflammatory cells [64,67]. Possibly, different fixation procedures may explain this discrepancy: rodent brains undergo intra-vital perfusion fixation, while the studies in man have to rely on specimens, which are fixed extra-corporally.

**Figure 2:**
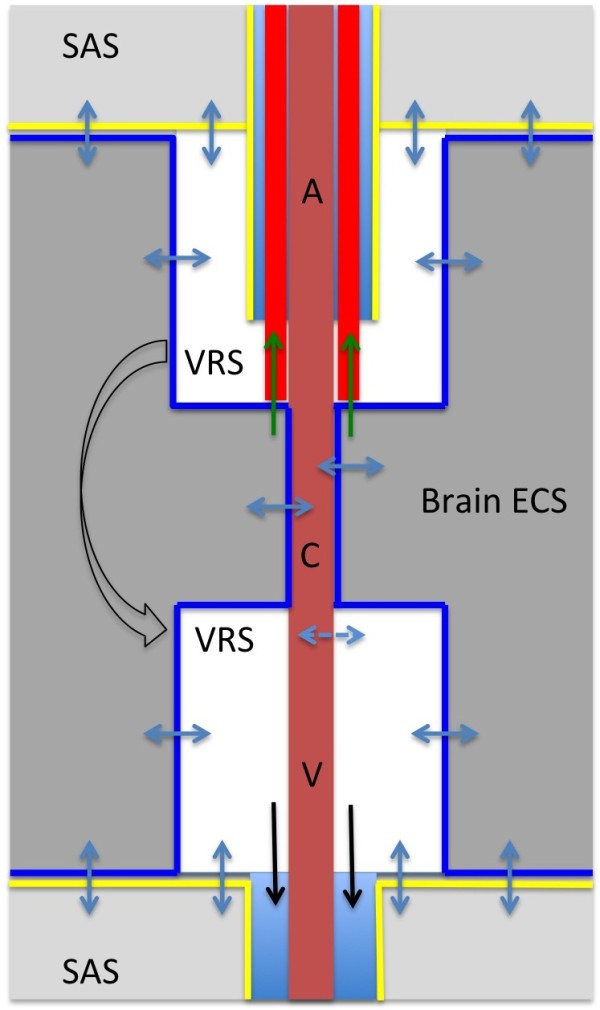
**Diagram representing fluid movements at the Virchow Robin space.** The complex anatomical structure of the Virchow Robin space (VRS) allows a bidirectional fluid exchange between the VRS and both the brain extracellular space (ECS) and the subarachnoid CSF space (blue arrows). Glial (blue lines) and pial (yellow lines) cell membranes enclose the VRS and control fluid exchange. Note, that it is a matter of debate whether the VRS represents an open fluid fill space (see text for discussion). Both experimental and clinical evidence indicate the existence of a pathway along the basement membranes of capillaries, arterioles, and arteries for the drainage of ISF and solutes into the lymphatic system (red lines and green arrows). It is unclear, whether the subpial perivascular spaces around arteries and veins (light blue) serve as additional drainage pathways. Also, the proposed glymphatic pathway connecting the arterial and venous VRS with the venous perivascular space (black arrows) is still a matter of debate. A: artery, V: vein, C: capillary, VRS: Virchow Robin space, SAS: subarachnoid space.

Although pial cell layers obviously separate the VRS from the cortical subarachnoid space, physiologically there is strong evidence indicating that fluid circulates along the VRS (Figure 2). Following the injection of horseradish peroxidase (HRP) into the lateral ventricles or subarachnoid space of anesthetized cats and dogs, light microscopic examination of serial brain sections has been performed utilizing a sensitive histochemical technique (tetramethylbenzidine incubation) [73]. The authors reported the distribution of tracer reaction product within the VRS and along the basal laminae around capillaries. The influx into these spaces was very rapid since the intraparenchymal microvasculature was clearly outlined 6 min after the infusion of HRP. Electron microscopy of sections incubated after 10 or 20 min of HRP circulation confirmed the paravascular location of the reaction product, which was also dispersed throughout the extracellular spaces (ECS) of the adjacent parenchyma. The rapid paravascular influx of HRP could be prevented by halting or diminishing the pulsations of the cerebral arteries by aortic occlusion or by partial ligation of the brachiocephalic artery. However, it should be noted that others were not able to reproduce these findings; Krisch *et al*. found no spread of HRP from the subarachnoid space into the VRS [70]. Also, another study reported that following microinjection into the VRS or the subarachnoid space of rats, tracers (e.g. India ink, albumin labeled with colloidal gold, Evans blue, rhodamine) remained largely in the VRS, the cortical subpial space and the core of subarachnoid trabeculae. Nevertheless, bulk flow of fluid within the VRS, around both arteries and veins, was suggested from video-densitometric measurements of fluorescently labeled albumin. However, the observed flow was slow and its direction varied in an unpredictable way [71]. Furthermore, it was shown that, following intracerebral injection, India ink particles concentrated in the VRS, but were then rapidly ingested by perivascular cells. Notably, very little movement of carbon-labeled perivascular cells and perivascular macrophages was seen after 2 years [74].

Since there is obviously at least some circulation of CSF into and out of the VRS, it raises the question how fluid and tracers could cross the pial membranes separating the VRS from the subarachnoid space. Ultrastructure studies have depicted the pial barrier as a delicate, sometimes single-cell layered structure [75]. There are considerable species differences: in the mouse the pial layer was found to be extremely thin, while in man its structure was significantly thicker [76]. Notably, in man the pial barrier was still described as a delicate yet apparently continuous layer of cells, which were joined by desmosomes and gap junctions but had no obvious tight junctions [77]. According to such morphological studies, it was recognized that the pia is not impermeable to fluids [61]. Since, in a similar fashion, the ependymal cell layers covering the inner (ventricular) surfaces of the brain are not connected by tight junctions [78], it was suggested that "CSF communicates with the ISF across the inner (ependymal) and outer (pial) surfaces of the brain" [61]. If one assumes that the flow within the VRS depends on the pulsatility of the arteries [73,79], hydrostatic forces may drive fluids and solutes across the pial membranes. However, while the VRS basically allows for the bi-directional exchange between CSF and ISF, no quantitative data are available that describe the extent and kinetics of such fluid movements. Although it has been shown that pial membranes between the PVS and the SAS could prevent the exchange of larger molecules, since tracer, following intraparenchymal injection, accumulated within the PVS but was not distributed into the cisternal CSF [80]. This observation is supported by clinical findings that following aneurysmal rupture in man, red blood cells are confined to the subarachnoid space, and do not enter the VRS [76].

It has also been shown both experimentally and clinically that the PVS and possibly more importantly intramural pathways between the basement membranes of the wall of arterioles and arteries provide drainage for the ISF and waste molecules of the brain. There is experimental evidence that the para-arterial drainage pathways are connected to the lymphatics of the exterior skull base [81,82]. Actually, solutes and fluid may be drained along the arteries from the brain interstitium via the VRS into the cervical lymphatics [81,83], reviewed by Weller [45]. Supporting this notion are the immunohistochemical and confocal microscopic observations that soluble fluorescent tracers (3 kD dextran or 40 kD ovalbumin) move from the brain parenchyma along the basement membranes of capillaries and arteries following its injection of into the corpus striatum of mice. This pathway may not serve for the transport of particles or cells, since fluospheres (diameter 0.02 μm and 1.0 μm) did not leave the brain but expanded the periarterial spaces and were locally ingested by macrophages. Clearance of solutes along this pathway could be prevented by cardiac arrest [83]. The finding that macromolecules may be drained from the brain via perivascular or intramural transport led to the notion that vessels and their pial sheaths act as ‘lymphatics of the brain’. These findings are clinically significant since based upon observations in patients with cerebral amyloid angiopathy, beta-amyloid is deposited in the vascular wall of arterioles and arteries. The deposition of insoluble amyloid may obstruct this drainage pathway and therefore impede the elimination of beta-amyloid and interstitial fluid from the brain in Alzheimer’s disease [82,84]. Interestingly, the extent of amyloid deposition is so prominent that it was suggested as a natural tracer for the peri-arterial drainage pathways [83]. The peri-arterial drainage of fluids and solutes has important implications not only in neurodegenerative diseases, but in addition in immunological CNS diseases, see for comprehensive reviews [45,85,86]. Similar to arteries, veins within the subarachnoid space possess a pial sheath forming a PVS [64]. As compared to arteries, it is less clear whether venous perivascular pathways serve as a drainage pathway for ISF and interstitial solutes. Notably, injections of tracers into the brain revealed no drainage along peri-venous channels unless there is disruption of flow in cerebral amyloid angiopathy when some tracer enter the peri-venous spaces [87]. However, recent findings [88] indicate a more significant contribution of the venous perivascular route for the drainage of ISF and solutes (see discussion below).

#### Interstitial fluid movement

Traditionally, movement of fluids through the brain interstitial space has been attributed to diffusional processes [89-91], which actually are slow because of the narrowness and tortuosity of the extracellular space of the brain (reviewed by [92] ). Today, it is commonly accepted that "the narrow spaces between cells within the neuropil are likely to be too small to permit significant bulk flow" [29]. A recent review discusses important clinical implications regarding CNS drug delivery [93]. As commented by others [45,94], our current understanding includes bulk flow mechanisms for the movement and drainage of ISF along white matter tracts and the perivascular spaces. Considering the cellular architecture of pia and ependyma, it also accepted that these cellular layers represent a diffusional barrier, which actually provides a communication between ISF and CSF [61]. Experimental evidence for the existence of bulk flow mechanisms was found after microinjection of tracer into the brain. Morphological studies revealed the VRS and the perivascular spaces as channels for fluid transport, but also revealed additional spaces between fiber tracts in white matter and the subependymal layer of the ventricle. Analysis of the kinetics of removal of three radiolabeled tracers from brain tissue (e.g. polyethylene glycols: 0.9 and 4 kD and albumin: 69 kD), provided evidence for the convection of ISF. These three test compounds differ in their diffusion coefficient by up to a factor of five but were cleared from brain according to a single exponential rate constant. This is consistent with removal by convection from a well-mixed compartment. For different regions of the brains of rats and rabbits, the ISF flow rate was estimated between 0.11 and 0.29 μl g brain^-1^ min^-1^[30,61], reviewed by [29]. Very recently it has been shown that astrocyte water transporters, i.e. aquaporin-4 (AQP4), contribute to interstitial brain water movement: in transgenic animals lacking AQP4, the interstitial drainage of tracer injected into brain parenchyma was significantly reduced [95].

### Towards a molecular understanding of brain water fluxes

The discovery of water transporters (‘water channels’) located at the end-feet processes of astrocytes has decisively improved our understanding of the physiology of the blood brain barrier and has led to the concept that large water fluxes take place continuously between the different compartments of the brain, i.e. the blood, CSF and ISF (reviewed in [96-98]). Interestingly, such extensive water movements were indicated by earlier radiotracer experiments. For example in 1952, following the intravenous injection of deuterium oxide a rapid distribution throughout all brain compartments was reported [99]. These data demonstrated water fluxes that greatly exceeded the contemporary estimated rates of CSF and ISF flow. As a result, the significance of this work was not fully appreciated. Recently the original data on the deuterium oxide half-life in different brain compartments has been used to calculate the respective CSF fluxes by applying MRI-based volume assessments of the ventricles, the subarachnoid space and the spinal CSF spaces. As result, CSF fluxes of more than 22 ml min^-1^ and a CSF turnover rate of more than 140 times a day were calculated. This is far greater than the traditional views of CSF physiology [100]. Of note, the permeability of deuterium oxide through AQP1 [101] and AQP4 [102] is similar to that of water.

#### Choroid plexus

CSF formation at the choroid plexus occurs in two stages: passive filtration of fluid across the highly permeable capillary endothelium and a regulated secretion across the single-layered choroidal epithelium. The choroidal epithelium forms a fluid barrier since tight junctions are expressed at the apical, CSF facing, cell membrane [103]. The rate of choroidal CSF formation is rather insensitive to osmotic and hydrostatic pressure changes in the CSF and therefore relatively independent of changes in intracranial pressure and plasma osmolarity. Hence, water transport across the choroid plexus epithelium cannot be explained simply by an osmotic mechanism (discussed in detail in [96]). Today there is agreement that choroidal CSF production is controlled by membrane transporters within the epithelium. Different transporters are expressed at the basolateral (plasma facing) and apical (CSF facing) membranes. Due to its high AQP1 expression, the apical membrane has high water permeability. In contrast to this, the basolateral membrane lacks significant AQP1 expression [104]. At the apical membrane a K^+^/Cl^-^ cotransporter is co-localized with the Na^+^/K^+^ -ATPase. Together, these transporters expel water from the cell into the CSF space. Little is known about the water transport at the basolateral membrane. There is a K^+^/Cl^-^ cotransporter, but its role is not yet well understood [96]. The molecular mechanisms of choroidal CSF production are comprehensively reviewed in [96,105,106].

#### Blood brain barrier

Traditionally the properties of the blood–brain barrier (BBB) are considered to be those of the capillary endothelium in brain. This endothelium contrasts with that elsewhere in the body by being sealed with tight junctions, having a high electrical resistance and a low permeability to polar solutes [89]. Early research unveiled ion channels and transporters capable of providing a net secretion of fluid, driven by Na^+^/K^+^ - ATPase, on the brain side of the barrier. Accordingly, the BBB was proposed as a secretory endothelium, which produces ISF [107]. Recent research has unveiled that the ‘barrier’ function of the BBB is in fact the result of highly regulated and complex cellular and molecular transport processes, which allow for the transport of water, solutes, larger molecules and even cells (reviewed by [108-110]). The modern understanding of BBB physiology was further improved by the discovery that cells surrounding the capillaries can control and modulate BBB functions. Considering the involvement of astrocytes, pericytes, microglia and even neurons, the BBB is better described as a ‘neurovascular unit’ [111]. The role of astrocytes is of utmost interest with respect to CSF physiology, since astrocyte end-feet have been shown to cover the entire capillary surface, leaving intercellular clefts of less than 20 nm [112]. The astrocytes, therefore, form an additional barrier surrounding the cerebral capillaries [98]. The role of astrocytes in brain water homeostasis is strongly supported by the finding that water transporting pores (i.e. the aquaporins) are localized in the end feet [113,114], reviewed by [97]. It is also important to recognize that contrary to earlier assumptions, the endothelial barrier carries no AQP4 transporters [115]. Instead, water may cross the endothelium by diffusion, vesicular transport and, even against osmotic gradients, by means of co-transport with ions and glucose (reviewed in [96]).

#### Aquaporins and other modes of water transport

The physiology of aquaporins (AQPs) and transporters in the brain has been comprehensively reviewed [96,98,116-118]. Here those aspects are discussed, which are relevant for the understanding of CSF circulation. Basically, in response to both passive osmotic and hydraulic pressure gradients, AQPs can transport water, solutes, and ions bi-directionally across a cell membrane. In comparison to diffusional transport, AQPs have significant biophysical differences. Diffusion is non-specific and low-capacity movement, whereas water channels like the AQPs provide rapid transport and have both a high capacity and a great selectivity for the molecules being transported [119]. As discussed below, that may be especially important for fluid exchange between ISF and CSF. More recent data in rodents have demonstrated that the precise dynamics of the astroglia-mediated brain water regulation of the CNS is dependent on the interactions between water channels and ion channels. Their anchoring by other proteins allows for the formation of macromolecular complexes in specific cellular domains (reviewed in [120]).

Currently, at least 14 different aquaporins have been identified [97,117]. At least six have been reported in the brain [121,122]: AQP 1, 4, 5 (specifically water permeable), AQP3 and 9 (permeable for water and small solutes) and AQP8 (permeable for ions) [116]. AQP4 is implicated in the formation/resolution of brain edema and in the clearance of K^+^ released during neuronal activity; AQP1 plays a role in cerebrospinal fluid (CSF) formation, and AQP9 may play a role in energy metabolism [97]. Positron emission tomography techniques for imaging of AQP4 in the human brain are currently being developed [123]. Structural and functional data suggests that the permeability of AQP channels can be regulated and that it might also be affected in brain pathologies (reviewed by [116,124]). As a result of the dynamic regulation, AQP channel permeability or AQP channel subcellular localization may change within seconds or minutes leading to immediate changes in the membrane permeability. Long-term regulation is mediated by changes in AQP mRNA and/or protein synthesis and/or degradation rate. These changes will alter AQP expression within hours or days. AQPs may be regulated under pathological conditions: For example AQP1 and AQP4 are strongly upregulated in brain tumors and in injured brain tissue [116], AQP5 is down-regulated during ischemia but up-regulated following brain injury [121].

Notably, AQP1 is expressed in vascular endothelial cells throughout the body but is absent in the cerebrovascular endothelium, except in the circumventricular organs [125]. As already discussed AQP1 is found in the ventricular-facing cell plasma membrane of choroid plexus epithelial cells suggesting a role for this channel in CSF secretion. In AQP1-null mice, CSF production was 20% less than in wild-type mice (0.38 ± 0.02 vs. 0.30 ± 0.01 μl min^-1^). Accordingly it was discussed that AQP1-facilitated transcellular water transport accounts for only part of the total choroidal CSF production. As a more controversial possibility, it was suggested that the choroid plexus may not be the principal site of CSF production and that extrachoroidal CSF production by the brain parenchyma may be more important [126,127]. The latter notion is supported by the observation that following its intravenous application, the penetration and steady concentration of H_2_^17^O is significantly reduced in ventricular CSF in AQP4 but not in AQP1 knockout mice. The authors concluded that AQP4 is more important for CSF production than AQP1 [122,128].

AQP4 is strongly expressed in astrocyte foot processes at the BBB, glia limitans of brain surface and VRS, as well as ventricular ependymal cells and subependymal astrocytes. Actually, it is expressed at all borders between brain parenchyma and major fluid compartments [97,113,114]. Therefore, the earlier view of exchange of ISF and CSF across ependymal and glial cell layers [129] may be in fact aquaporin-mediated water transport across these membranes [130]. AQP4 is also localized in astrocyte end feet at the perisynaptic spaces of neurons and is found in the olfactory epithelium [97]. The precise subcellular distribution of AQP4, i.e. in the astrocyte foot processes, is regulated by its association with the dystrophin glycoprotein complex, including dystrophin, beta-dystroglycan, and syntrophin [131,132]. In mice lacking alpha-syntrophin, astrocyte AQP4 is displaced, being markedly reduced in the end feet membranes adjacent to the blood vessels in cerebellum and cerebral cortex, but present at higher than normal levels in membranes directly facing the neuropil [131]. Others reported that the deletion of alpha-syntrophin causes a 50% loss of AQP4 from the cortical membrane as compared with a 90% loss at the perivascular membrane [133]. A similar effect on AQP4 localization is observed in dystrophin-null mice [134]. AQP4 has been suggested to interact with the inwardly rectifying K^+^ channel Kir4.1 [135]. Since Kir4.1 is also associated with the dystrophin glycoprotein complex the pattern of the subcellular distribution of AQP4 and Kir4.1 in astrocytes is very similar [136].

AQP4 is involved in water movements under pathological conditions (see for details [97,125,137,138]). There is agreement that AQP4-null mice have reduced brain swelling and improved neurological outcome in models of (cellular) cytotoxic cerebral edema including water intoxication, focal cerebral ischemia, and bacterial meningitis. However, brain swelling and clinical outcome are worse in AQP4-null mice in models causing a disruption of the BBB and consecutive vasogenic edema. Impairment of AQP4-dependent brain water clearance was suggested as the mechanism of injury in cortical freeze-injury, brain tumor, brain abscess and hydrocephalus [125]. In hydrocephalus produced by cisternal kaolin injection, AQP4-null mice demonstrated ventricular dilation and raised intracranial pressure, which were both significantly greater when compared to wild-type mice [139].

It is a matter of ongoing research whether AQP4-mediated brain water movement is relevant under physiological conditions. Considering only the pattern of AQP4 expression at the borders between the brain and CSF compartments, it has been suggested that AQP4 facilitates or controls the flow of water into and out of the brain [98]. However, how aquaporins modulate CSF/ISF circulation and whether they impact fluid flow in extracellular pathways within the tightly packed neuropil is only poorly understood. Since AQP4 is also expressed at astrocytic end feet near the perisynaptic spaces, a putative role for astrocytes and AQP4 for K^+^ homeostasis during neuronal activity has been postulated (reviewed by [97]). AQP4 deletion is associated with a sevenfold reduction in cell plasma membrane water permeability in cultured astrocytes [140] and a tenfold reduction in BBB water permeability in mouse brain [141]. However, AQP4 deletion was found to have little impact on CSF dynamics (reviewed by [106]). In AQP4-null mice unaltered intracranial pressure and compliance were found [142]. Furthermore, no changes in ventricular volume or anatomical features of two different AQP4-null mice strains were reported [143]. However, others observed smaller ventricular sizes, reduced CSF production and increased brain water in AQP4-null mice [144]. Considering that the deletion of AQP4 has only little or modest *in vivo* effects, the current view is that, under normal physiological conditions, AQP4 is not needed for relatively slow water movement conditions [97]. However, the minimal impact of AQP4 deletion on CSF physiology may be explained by the fact that AQP4 deletion reduces both ISF/CSF formation and absorption. Mice in which a conditional knockout was driven by the glial fibrillary acidic protein promoter, showed increased basal brain water content. In these animals the extracerebral AQP4 function is preserved but AQP4 is eliminated in cells that express the GFAP promoter, i.e., astrocytes and ependyma. After systemic hypo-osmotic stress by intraperitoneal water injection, those mice showed a 31% reduction in brain water uptake. It was concluded that the glial covering of the neurovascular unit limits the rate of brain water influx as well as the efflux [115].

**Figure 3:**
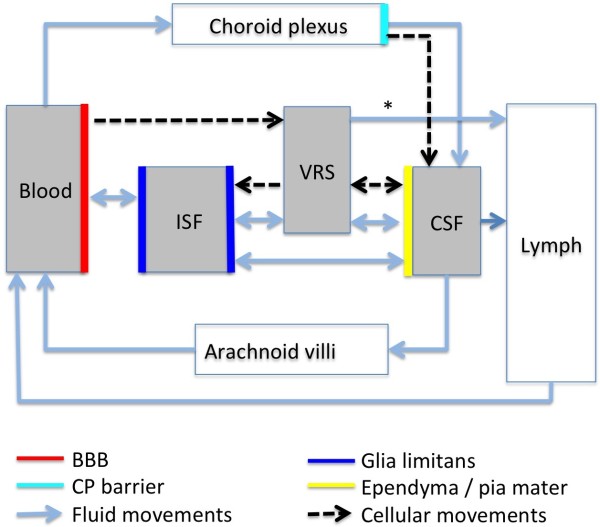
**Diagram of the CSF "Circulation".** This diagram summarizes fluid and cellular movements across the different barriers of the brain compartments (blood, interstitial fluid, Virchow Robin space, cerebrospinal fluid space comprising the cerebral ventricles, basal cisterns and cortical subarachnoid space). Aquaporins and other transporters control the fluid exchange at the glial, endothelial, and choroid plexus barrier. At the glial, endothelial, and pial barrier bi-directional flow may generate either a net in- or outflux, providing fluid exchange rates, which surpass the net CSF production rate by far. The choroid plexus is the only direct connection between the blood and the CSF compartment. Major portions of brain water are drained into the cervical lymphatics from the VRS (including its capillary section) via intramural arterial pathways (asterisks) and from the CSF space (via perineural subarachnoid space of cranial nerves). The capillary and venular endothelium may contribute to brain water absorption. Blood borne inflammatory cells may enter the brain via VRS venules or via CP. Fluid movements at the barriers are driven by osmotic and hydrostatic gradients or by active transporter processes. Fluid movements into and out of the VRS depend on respiratory and cardiac pressure pulsations.

It is now widely accepted that water moves across the endothelium by simple diffusion and vesicular transport, and across the astrocyte foot process primarily through AQP4 channels (reviewed by [98]). In addition, a variety of endothelial water-transport proteins expressed in one or both of the cell membranes (luminal or apical), provide co-transport of water along with their substrates even independently of osmotic gradients. Especially the glucose transporter GLUT1 and the Na^+^/K^+^/2Cl^-^ cotransporter, NKCC1, may contribute significantly to transendothelial water transport (reviewed by [96]). The identification of non-aquaporin water transporters located at the endothelium was a major contribution to the understanding of water transport across the neurovascular unit (not just the astrocyte or endothelial barrier). It is important to recognize that all these transport mechanism are bi-directional and represent a dynamic process. This implies that large water fluxes may take place continuously, although the net flow may be small. This would explain the fast and extensive passage of deuterium oxide from blood to brain [99]. As a process independent of net flow, the finding could be understood as a result of a dynamic bidirectional mixing of water between the blood, ISF and the CSF compartments. The bidirectional transport could also generate net-flux. Actually, the neurovascular unit may not only be involved in the production but also in the absorption of CSF and ISF. This is suggested by recent experiments in which tritiated water was infused into the ventricle of cats. During a three-hour infusion, the concentration in blood sampled from the cerebral venous sinuses rapidly increased up to 5 times higher than in samples of cisternal CSF and arterial blood. However, following the infusion of ^3^H-inulin, the cisternal concentration increased sharply during the observation period of three hours. At the same time venous and arterial concentrations were near background activity. It was concluded that ^3^H-water, but not ^3^H-inulin, is absorbed from brain ventricles into periventricular capillaries, which eventually drain in the venous sinuses [145]. Figures 2 and 3 illustrate that aquaporins, associated with astrocytes in the glial and ependymal cell layers, may control brain water movement around the Virchow Robin space and across the brain compartments.

#### Magnetic resonance flow studies

Phase-contrast magnetic resonance imaging (MRI) can provide quantitative blood flow velocity information in humans [146]. It was applied to the study of CSF flow along the aqueduct, a small canal connecting the third and fourth cerebral ventricles [147,148]. Advanced phase-contrast MRI, the cine phase-contrast technique yields quantitative flow information by synchronizing the acquisition of the images to the cardiac cycle [149]. Eventually, these MRI techniques may be applied to assess the heartbeat related stroke volume of CSF, from which the CSF net flow along the aqueduct may be calculated [150]. Applying these techniques, the normal aqueduct flow has been measured many times in adults with flow rates ranging from 0.304 to 1.2 ml min^-1^[151-155]. Based upon these data, the average normal flow in healthy adults was suggested to be 0.77 ml min^-1^ in the craniocaudal direction [7]. Hence, CSF flow measured by MRI exceeds the customarily assumed choroidal CSF production rate by two fold. Findings showing a reversed (caudocranial) flow of CSF along the aqueduct are even more puzzling. A reversed flow of 0.41 ± 0.51 ml min^-1^ was reported in children younger than two years [7]. Furthermore, a reversed flow was reported in adult patients suffering from normal pressure hydrocephalus: mean stroke volume in the control group was 30.1 ± 19.8 μl/cycle (craniocaudal direction), while that in the NPH group it was -63.2 ± 49.0 μl/cycle (caudocranial direction) [156]. In NPH patients, similar observations were reported by others [152]. Technical limitations of the MRI flow measurements must be considered before interpreting these MRI data that are not congruent with the traditional understanding of CSF physiology. Thus it was pointed out that the evaluation of the flow void is subjective and highly dependent on the acquisition parameters used, as well as on the technical characteristics of the MR imaging systems (e.g. gradient strength) [147]. Unfortunately, there is no class A evidence reported, which would clarify these conflicting data. Appropriate clinical studies would be important. Also, MRI techniques may be used to study interstitial water movement: diffusion-weighted MRI provides a quantitative parameter, i.e. the apparent diffusion coefficient (ADC), which is thought to reflect water mobility in brain tissues. Applying this technique it was shown in the rat brain that reducing AQP4 protein expression with small interfering RNAs (siRNAs) by 27% caused a 50% decrease of the ADC [157].

### Current research

There are numerous limitations of the early experiments that form our classical understanding of CSF physiology. Recent progress in neuroanatomy, molecular and cellular biology, and neuroimaging challenge the traditional model. The pillars of the classical model, i.e. CSF production at the choroid plexus, directed bulk flow and absorption across the arachnoid villi are currently being questioned. More recent experimental and clinical data have caused a growing number of researchers to reach the consensus that ISF and CSF are mainly formed and reabsorbed across the walls of CNS blood capillaries, which implies that there is no need for a directed CSF circulation from CP to the arachnoid villi. Eventually, a number of "unequivocal" findings, often more than 100 years old and still governing the customary understanding of CSF physiology, must be revised [7,9,10,88,95,98,122,158,159].

However, the novel concepts are also challenged mainly by the lack of validated supporting data. For example, Klarica *et al.* failed to reproduce the historical experiments of Dandy [13], since no circulation of CSF was found along a plastic cannula introduced into the aqueduct of cats [16]. Subsequent experiments demonstrated that the CSF pressure is not increased during the first hours after the occlusion of aqueduct of Sylvius [160]. Since they furthermore showed that following its intraventricular injection radioactive water is almost completely absorbed in the ventricles and does not reach the basal cisterns [145], they concluded that the choroid plexus is not the major site of CSF production and that no directed CSF circulation according to the classical understanding exists. Instead they proposed a model that assumes CSF production and absorption occurs at the level of the capillaries [10]. Considering the existence of CSF flow along the aqueduct as shown by MRI flow studies, others recognized that a model assuming CSF flow exclusively at the capillary bed is deficient [7]. Furthermore the view of Klarica *et al*. that CSF production and absorption just depend on hydrodynamic and osmotic gradients is not substantiated by current cellular and molecular biology findings. In fact, the proposed model does not consider the complex regulation of water movement between the brain compartments as discussed above. Finally, as in the original experiments of Dandy, the experiments of Klarica *et al.* may be criticized since they are surgically invasive and therefore results should be interpreted cautiously.

There are similar concerns with the most recent publications of Nedergaard and her group. In a series of experiments, fluorescent tracers of different molecular weight were injected into the cisterna magna of mice [95]. Applying two-photon laser scanning microscopy through a closed cranial window, the distribution of tracers could be observed 60–120 μm below the cortical surface. The experiments showed a rapid increase of fluorescence within the Virchow Robin space around the arterioles. Fluorescent tracer was subsequently found within the brain interstitium and later around the venules. Histological examination 30 minutes after cisternal fluorescent tracer injection revealed that larger molecular tracer (FITC-d2000, 2000 kD) was confined to the VRS, while smaller molecular weight tracer (TR-d3, 3 kD) was concentrated in the VRS and also entered the interstitium. Investigating AQP4-deficient mice with the same experimental techniques, the authors found significantly less fluorescence within both, the VRS around the arteries and in the brain interstitium [95]. Considering the temporospatial occurrence of fluorescence, the authors deduced the existence of a directed flow of CSF from the subarachnoid space along the arteries and arterioles into the VRS, from here into the brain interstitium, and finally from the brain into the VRS around the venous vessels. Since the authors showed in AQP4 deficient mice that, following its interstitial injection, the clearance of soluble amyloid beta was significantly reduced, they concluded to have discovered an unknown system for the clearing of interstitial protein waste [88,161]. Assuming the PVS to serve as lymphatics of the brain (a notion which was conceptualized already in 1968 by Foldi [162]) and considering the involvement of astrocytes and their aquaporins the authors coined the term "glymphatics" to describe the system [95].

It should be noted that the glymphatic concept assumes transport from the SAS INTO the parenchyma along periarterial pathways. This notion is supported by previous findings of Rennels *et al.*[73]. However, as already discussed above, especially the work of the groups of Cserr [94] and Weller [45,70] support the view that the periarterial flow provides a drainage OUT of the parenchyma. Furthermore the findings of Nedergaard’s group are not consistent with previous work applying real-time video-densiometric techniques. Such experiments have depicted the movement of tracers within the VRS to be sluggish and the direction of flow varying in an unpredictable manner [71]. Currently, it is difficult to come to final conclusions about the direction of perivascular CSF flow. This is a complex research topic with difficult, technically challenging experiments not easily replicated among the different groups.

Regarding the glymphatic concept, one may criticize that it is based on two-photon laser scanning microscopy applying a 1 min scan time, which was optimized to acquire a 240 μm stack at 20 μm intervals. This appears to be a limitation of the scanning technique in terms of temporal resolution. It seems important to provide data with higher frequency imaging to clarify the direction of perivascular flow [71]. Furthermore, with the published data, it can’t be excluded that the observed fluorescence around subarachnoid arteries may reflect the nonspecific binding of dextran to the basement membranes of arteries [87]. Considering these criticisms and the point that the glymphatic concept represents a fundamental revision of the current understanding of CSF physiology, we feel that the concept needs to be substantiated by comprehensive ultrastructural investigations. Also studies in other species are warranted.

A subsequent publication of Nedergaard’s group reports the exciting possibility of an increased clearance rate of brain waste along the glymphatic pathway during sleep [163]. This conclusion was derived from *in vivo* two-photon laser scanning microscopy, fluorescent microscopy and measurements of the ISF volume comparing awake, asleep and anaesthesized animals. However, again, this is a very complex study design possibly prone to experimental errors: investigating animals with multiple brain catheters and fixated in a stereotactic or microscopic holder, one may assume that awake animals are under massive stress and may fell asleep just because of exhaustion. Although microdialysis was used to measure norepinephrine levels as a gauge for stress levels and norepinephrine did not increase in the experiments, important stress parameters may differ between the experimental groups, i.e. arterial blood pressure, venous blood pressure, stress hormone blood levels, heart and respiratory rate and blood gases. The fact that none of these parameters was recorded during the experiments is a major drawback, since each of the parameters may alter cerebral blood flow, cerebral blood volume, intracranial pressure and even the perivascular pump [79]. Each of these parameters may in turn influence ISF and CSF circulation and the width of the interstitial space.

In spite of this criticism, the observation that astrocytes are involved in the clearance of interstitial waste molecules including soluble amyloid is exciting. In this regard the experiments of Iliff *et al*. [95] revive the work of Cserr [30], who showed that bulk flow mechanisms contribute to the clearance of tracers injected into the brain interstitium. Confirmatory evidence of the impact of aquaporins on ISF regulation has been independently reported by others [115].

## Conclusions

The new findings do not render all of the previous, sometimes historical, work invalid. However, CSF researchers and clinicians have to recognize that much of the earlier findings need to be re-interpreted.

For example, the discovery of aquaporins and other water transporters, all highly selective just for water molecules, implies that the extent of water exchange across the barriers may be heavily underestimated by the classical flow studies [30,49]. The tracers, used in the classical experiments, were always larger than water molecules and therefore could not be a substrate for the water transporters. Also, the notion that osmolality does not impact CSF absorption [2,33] or that CSF absorption into capillaries requires a hydraulic pressure gradient, which would cause the collapse of the vessel [47], needs to be reconsidered. Even more puzzling, the notion of directed bulk flow movement of CSF, i.e. flow from the choroid plexus into the ventricles and along the cisterns and the subarachnoid space across the arachnoid villi back into the blood, must be questioned. As already suggested by others [9,164], the novel findings indicate that CSF circulation is much more complex, a combination of directed bulk flow, pulsatile to and fro movement, and continuous bi-directional fluid exchange at the blood brain barrier and the cell membranes at the borders between CSF and ISF spaces. Ongoing research emphasizes the role of lymphatic pathways for the drainage of ISF and CSF [45]; the observation that astrocytes and their aquaporins drive lymphatic drainage may open a new field of research [95]. The new insights into the physiology of CSF circulation may have important clinical relevance for example for the understanding of neurodegenerative and immunological diseases of the brain [45,161]. Also, opposing the classical view that drugs injected into the CSF space will be washed out within short time without targeting the brain [5], recent findings demonstrate that drugs, following intrathecal application, may very well be transported throughout the entire brain [165].

## Competing interests

The authors declare no financial or non-financial competing interests.

## Authors’ contributions

TB drafted and wrote the manuscript. ES, JM and PK commented and revised the manuscript. All authors have read and approved the final version of the manuscript.
